# High‐Resolution Imaging via UHF‐US–Based 3D Reconstruction in a 3D In Vivo Tumor Model

**DOI:** 10.1155/ijbi/8763250

**Published:** 2026-10-26

**Authors:** Andreas Ettner-Sitter, Jonas Kuenzel, Mia Heidenreich, Lina Mahl, Luise Pohnert, Dharini Srinivasan, Eric Pion, Robert Bauer, Dieter Fuchs, Benedikt J. Wagner, Christian Gröger, Felix Steger, Oliver Kölbl, Thiha Aung, Christina Hackl, Silke Haerteis

**Affiliations:** ^1^ Institute for Molecular and Cellular Anatomy University of Regensburg Regensburg Germany uni-regensburg.de; ^2^ Department of Surgery University Hospital Regensburg Regensburg Germany uniklinikum-regensburg.de; ^3^ TUM School of Life Sciences Technical University of Munich Freising Germany tum.de; ^4^ Faculty of Applied Healthcare Science Deggendorf Institute of Technology Deggendorf Germany; ^5^ PIUR IMAGING GmbH Vienna Austria; ^6^ FUJIFILM VisualSonics Inc. Amsterdam the Netherlands visualsonics.com; ^7^ Department of Radiotherapy University Hospital Regensburg Regensburg Germany uniklinikum-regensburg.de

**Keywords:** 3D tumor imaging, chorioallantoic membrane (CAM), high-frequency ultrasound, in vivo model, pancreatic cancer, patient-derived xenografts (PDX)

## Abstract

The chorioallantoic membrane (CAM) model resembles a preclinical in vivo platform for the engraftment of different tumors and is especially well‐suited for the development and evaluation of novel cancer therapies. Due to the CAM′s extensive vascular network, it can sustain engrafted human tumor tissue, which can be used for therapy testing and analysis throughout the experimental process. Although numerous studies have demonstrated successful tumor engraftment and vascular integration into the CAM′s blood vessel network, the accurate and sequential measurement of tumor volume remains a significant challenge. Current imaging modalities, such as positron emission tomography/computed tomography, magnetic resonance imaging or three‐dimensional (3D) digital microscopy are limited by cost, complexity, and potential harm due to injected agents or radiation. This study introduces an innovative imaging approach by utilizing ultrahigh frequency (UHF) ultrasound combined with 3D ultrasound reconstruction of pancreatic ductal adenocarcinoma (PDAC) on the CAM. By utilizing ultrasound frequencies of up to 71 MHz, two‐dimensional (2D) imaging of PDAC tumors was transformed into comprehensive 3D reconstructions. This innovative approach provides a more detailed tissue resolution and enables noninvasive, longitudinal volumetric monitoring of tumor growth in vivo, and in principle provides a platform for future studies assessing angiogenesis and therapy response. In this study the accuracy of 3D UHF ultrasound was tested by comparing it with established imaging techniques such as 3D digital microscopy. In summary, the 3D ultrasound reconstruction resembles a more versatile noninvasive alternative for in vivo tumor volume measurement. This advanced imaging approach adds a valuable methodology for the investigation of tumor biology in the CAM model by providing noninvasive volumetric and morphological characterization of engrafted tumor tissue, thereby linking preclinical research and clinical applications.

## 1. Introduction

The chorioallantoic membrane (CAM) model has recently gained a lot of interest by researchers as a cost‐efficient and versatile preclinical in vivo platform for the development and testing of potential cancer therapies [1, 2]. The CAM resembles an extraembryonic membrane that is formed during embryogenesis within fertilized chicken eggs and has a dense vascular network, which facilitates gas exchange comparable to a human placenta. Multiple human tumor samples have been successfully engrafted onto the CAM and remained vital for investigation due to the dense vascular network that supplies nutrients and oxygen in addition to an underdeveloped immune system [1–3]. During CAM‐experiments, therapeutic agents can be administered either topically or intravascularly and their effects on tumor angiogenesis, tumor volume and tumor metabolism can subsequently be monitored using various methods [4]. These include laser speckle contrast analysis (LASCA), ultrahigh frequency (UHF) ultrasound (US) and immunohistochemistry, which enable the visualization of blood flow and tumor volume changes [5–8].

A recent study further expanded this application by integrating patient‐derived xenograft (PDX) techniques through the generation of tumor spheroids from circulating cancer stem cells (cCSCs) of pancreatic ductal adenocarcinoma (PDAC) and their engraftment onto the CAM [9]. However, reproducible and intraexperimental in vivo measurement of tumor volume during the application and testing of new therapies remains a difficult challenge to overcome when using the CAM model. Many current imaging methods such as positron emission tomography (PET)/computed tomography (CT), three‐dimensional (3D) digital microscopy and magnetic resonance imaging (MRI) are often complicated, expensive and potentially harmful due to radiation or injected contrast agents [6, 10, 11].

In this study, we present a novel and noninvasive 3D imaging method based on the 3D reconstruction of PDAC tumors that were measured with UHF‐US on the CAM. This resembles a significant advancement to the previously published study that showcased the use of UHF‐US as a two‐dimensional (2D) imaging technique for the monitoring of kidney cysts engrafted onto the CAM [7]. By utilizing US frequencies of up to 71 MHz, we were able to substantially enhance tissue resolution and differentiation of the PDAC from the CAM in a noninvasive manner [7]. Since conventional 2D US falls short of providing comprehensive and detailed volumetric information, we added a handheld 3D motion sensor (PIUR IMAGING) for the 3D reconstruction of 2D US imaging of PDAC on the CAM.

The aim of the study was the development of a novel UHF‐US 3D imaging modality for PDAC analysis in the CAM model. The proposed methodology outperformed 2D US imaging as it enabled rapid in vivo 3D visualization of the observed PDAC tissue and thereby captures short‐term changes of the tumor volume. The results were validated with conventional imaging methods such as 3D digital microscopy. The study positions 3D US as a versatile imaging methodology, which offers distinct advantages such as noninvasiveness, high spatial resolution, and in vivo monitoring. Furthermore, the model also complies with the 3R‐principles (replacement, reduction, and refinement) for the improvement of living conditions of animals that are used for scientific purposes.

## 2. Materials and Methods

### 2.1. Patient Tissue

PDAC tissue samples were obtained from patients diagnosed with PDAC who underwent surgical resection at the Department of Surgery University Hospital Regensburg. Prior to surgery, all eligible patients provided informed consent for participation in the research project in accordance with the ethical standards outlined in the World Medical Association Declaration of Helsinki–Ethical Principles for Medical Research Involving Human Subjects. The study received approval from the ethics committee of the University Hospital Regensburg (Ethics Approval No. 20‐1989‐101). An approval of the ethics committee of the University Hospital Regensburg was also previously obtained (Ethics Vote 16.05.2022 Number 20‐1989‐101).

### 2.2. Hardware

#### 2.2.1. 3D In Vivo Model

The CAM model was carried out according to established protocols previously described by our research group [5, 7] (Figure 1). Fertilized chicken eggs were obtained from a certified supplier and initially cleaned of surface contaminants. The eggs were then transferred to a ProCon egg incubator (Table 1) (Grumbach, Asslar, Germany) and maintained at 37.8 °C with a relative humidity of 63%. To ensure physiological embryonic development and to prevent adhesion of the embryo to the eggshell, the eggs were mechanically rotated during the first 4 days of incubation. On Day 4 of embryonic development, access to the CAM was established by creating two openings in the eggshell. First, the air chamber, typically located at the blunt or pointed pole of the egg, was identified using a gooseneck lamp. After stabilizing the shell, a small window of approximately 0.1 cm^2^ was carefully opened using fine scissors and forceps to puncture the air chamber. This opening was subsequently sealed with surgical adhesive tape (Leukosilk, BSN medical, Hamburg, Germany), generating a slight negative pressure that caused the CAM to detach from the shell membrane.

**Figure 1:**
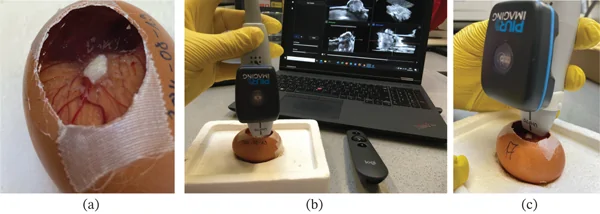
Experimental setup. (a) PDAC tumor tissue engrafted on the CAM. (b) Device setup. The 3D sonography transmission video box is connected to FUJIFILM VisualSonics′ Vevo MD via HDMI and to the processing laptop via WiFi. The 3D sonography sensor is magnetically attached to the transducer. The scans can be recorded and stopped via remote control. (c) Side view of the transducer and connected 3D sensor in a tumor‐engrafted CAM.

**Table 1:** Equipment and consumables used.

Device/material name	Reference
Canon Aquilion LB TSX‐201A	Canon Medical Systems GmbH, Neuss, Germany
Digital Microscope VHX‐7000 series	Keyence Deutschland GmbH, Neu‐Isenburg, Germany
Elekta Agility	Elekta Instrument AB, Stockholm, Sweden
Incubator	ProCon automatic systems GmbH & Co. KG, Mücke/Sellnrod, Germany
Leica M205 A	Leica Microsystems GmbH, Wetzlar, Germany
Leukosilk S	BSN Medical GmbH, Hamburg, Germany
Phoenix v‐tome‐x s	GE Sensing & Inspection Technologies GmbH, Wunstorf, Germany
PIUR tUS Infinity (Infinity video box, PIUR sensor)	PIUR IMAGING GmbH, Vienna, Austria
Vevo MD ultrasound system	FUJIFILM VisualSonics Inc., Toronto, Canada

All devices and consumables used in this study are listed in Table 1.

A second window of approximately 1 × 1 cm was then created along the longitudinal axis of the egg to expose the CAM surface. This window was temporarily sealed with a removable strip of Leukosilk (Table 1) to protect the membrane from dehydration and microbial contamination. On Day 8 of embryonic development, human tissue was cut into small pieces and grafted onto the CAM; the eggs were then returned to the incubator and maintained for an additional 7 days.

#### 2.2.2. 2D Ultrasonography

To perform 2D US the Vevo MD Imaging System (FUJIFILM VisualSonics Inc., Toronto, Canada) with a 71‐MHz transducer was used by hand with a steady motion to monitor PDAC tumors on the CAM. UHF‐US provides the highest resolution achievable in medical US with B‐mode imaging at 30‐*μ*m resolution and a maximum penetration depth of 10 mm [12]. The resulting 8‐s clips that captured the entire tumor tissue engraftment were analyzed via Vevo LAB software (Table 2) (FUJIFILM VisualSonics).

**Table 2:** Software and consumables used.

Software name	Reference
GraphPad Prism 9.2.0	GraphPad Software, San Diego, United States
Leica Application Suite 4.13.0	Leica Microsystems CMS GmbH, Wetzlar, Germany
Monaco 5.51	Elekta AB, Stockholm, Sweden
VGStudio MAX 2.2.3	Volume Graphics GmbH, Heidelberg, Germany
Vevo LAB software v3.2.1 #182	FUJIFILM VisualSonics, Inc. Toronto, Canada
PIUR tUS Infinity software	PIUR IMAGING GmbH, Vienna, Austria

All software used for image acquisition, data analysis and statistical analysis is listed in Table 2.

#### 2.2.3. 3D US

The Vevo MD system combined with the PIUR Infinity 3D US platform (PIUR IMAGING GmbH, Vienna, Austria) referred to as “3D sonography” enables 3D reconstruction of 2D US images. This method was used to quantify volumes measured with the 2D US with the Infinity sensor mounted to the transducer to capture 3D volumes and a stationary mount (Figure S1). To monitor tumor growth and vascular development, US imaging was conducted on Day 3 of engraftment. Prior to image acquisition, 1 mL of sterile sodium chloride (NaCl) solution was applied to the tissue surface to ensure optimal acoustic coupling and was removed immediately after scanning. Additionally, the window in the eggshell was enlarged to create an appropriate size for the US transducer. Image acquisition was performed in B‐mode with a spatial resolution of 30 *μ*m and a maximum penetration depth of 10 mm. For each imaging session, 8‐s video sequences were recorded with steady motion in one direction. Data analysis was conducted semiautomatically via “Tomographic Ultrasound” software (PIUR IMAGING GmbH). Reproducibility was evaluated through repeated scans and each scan was analyzed three times. Subsequently, 3D volume meshes were exported and processed in Blender software (v. 4.2.0) to quantify the area and volume via the 3D Print Toolbox (Figure S2).

### 2.3. Software

### 2.4. 3D Volume Measurements

#### 2.4.1. 3D Digital Microscopy

The Keyence VHX‐7000 (Table 1) (KEYENCE Deutschland GmbH, Neu‐Isenburg, Germany) is a reflected‐light digital microscope that reconstructs surface topography based on the “Depth from Defocus” (shape‐from‐focus/focus‐variation) principle. During image acquisition, the objective is moved through a range of focal heights, and for each lateral position, the height of maximum local sharpness is used to generate a 3D surface, a so‐called height map. Using the integrated analysis software, the tumor/sphere base and measuring range were manually defined, after which tissue volume was calculated from the reconstructed *x*‐, *y*‐, and *z*‐dimensional axis. This digital microscopy approach can potentially replace caliper‐based methods and enables volume measurements of patient tissue in the CAM model both before and after engraftment. Here, volumes of PDAC tissue, renal cystic tissue and phantom spheres of predefined volume were measured either ex vivo before engraftment, after excision or in vivo on Day 3 of cultivation. However, in vivo measurements remain challenging because of distortions caused by light reflections and the inability to measure tumor growth underneath the CAM [6].

#### 2.4.2. CT Measurements

CT‐based volume measurements of renal cystic tissue engrafted on the CAM were conducted based on scans of the whole egg recorded with a clinically approved CT scanner based on the established protocol (Figure S3) [13]. CT scans were acquired at 80 kV and an effective tube current‐time product of 436 mAs (tube current 300 mA, rotation time 1 s), with a collimation of 0.5 × 16 and a reconstructed slice thickness of 1 mm, over a scan length of 51 mm. The same 10 eggs were imaged at both time points; eggs were stored in the incubator between scans and vitality was confirmed prior to the second acquisition to ensure that survival of the embryo was maintained across the imaging interval.

To enable reliable visualization of soft tissues despite the small size of the CAM model, a dedicated scanning protocol was developed in collaboration with the CT device manufacturer and specifically optimized for soft tissue imaging. The selected acquisition parameters were chosen to maximize soft tissue contrast and spatial resolution, allowing a detailed assessment of engrafted tissue samples on the CAM. CT scans were performed 3 days postengraftment and, depending on tissue‐specific requirements, were acquired in either spiral or sequential mode. Although clinical CT systems are primarily designed for the imaging of human anatomy, this approach enabled visualization of relatively small tumor samples engrafted on the CAM; however, decreasing sample size limited the accuracy of structural delineation and thereby affected the precision of volumetric measurements.

Prior to each scan, two preliminary topogram images were acquired in anteroposterior and lateral orientations to accurately define the scan range and ensure precise coverage of the region of interest while minimizing unnecessary radiation exposure to surrounding embryonic structures. Scan durations were limited to a few minutes per egg, and all imaging procedures were conducted in compliance with clinical safety guidelines, with radiation doses remaining within acceptable limits for embryonic tissue.

Following image acquisition, raw CT data were processed using the integrated reconstruction software of the CT system. Various reconstruction algorithms and parameter adjustments were applied to enhance image sharpness and overall quality. Specific reconstruction filters and settings were established to optimize soft tissue contrast for the in vivo CAM model, including applications involving renal cysts and pancreatic tumor tissue.

This imaging protocol was specifically adapted for the longitudinal evaluation of transplanted renal cysts in fertilized chicken eggs. Ten eggs were analyzed at two defined time points, shortly after inoculation and immediately prior to tissue excision, to determine cyst volumes and monitor tissue growth over time.

### 2.5. Statistical Analysis

All statistical analyses were performed in GraphPad Prism 9.2.0 (Table 2) (GraphPad Software, San Diego, California, United States) and using R 4.6.1 (R Foundation for Statistical Computing, Vienna, Austria). Data are presented as mean ± standard deviation (SD) unless stated otherwise. Throughout, *n* denotes the number of independent biological replicates (individual CAM models or individual phantom spheres); repeated analyses of the same scan are reported separately as technical replicates (each measurement was repeated two times and averages were calculated prior to analysis). Phantom volumes are reported in cubic millimeter (1 *μ*L = 1  mm^3^). Agreement between 3D US or 3D digital microscopy and the reference values of phantoms of defined volume (50, 100, and 200 mm^3^; *n* = 4–5 spheres per category) was assessed by two separate Bland–Altman analyses on all individual paired measurements, reporting mean bias and 95% limits of agreement (LoA) (bias ± 1.96 × SD). For each category, volumetric measurements obtained by 3D UHF‐US or 3D digital microscopy were compared using exploratory Wilcoxon signed‐rank tests to assess method‐related differences. Paired renal‐cyst volumes obtained by CT and digital microscopy were compared using a two‐tailed Wilcoxon matched‐pairs signed‐rank test (*n* = 10). A *p* value < 0.05 was considered significant. Exact *n* and, where applicable, *p* values, including nonsignificant comparisons, are reported in the figure legends.

## 3. Results

### 3.1. Tissue Engraftment in the CAM Model

PDAC tissue was obtained from patients diagnosed with PDAC who underwent surgical resection at the University Hospital Regensburg and engrafted on the CAM. PDAC samples were engrafted immediately after surgical resection on the same day (Figure 1a). Histological analysis showed vital tissue after 7 days engraftment with enhanced vascular branching surrounding the PDAC tumor, with solid or dispersed growth and large polymorphous PDAC cells surrounding empty lumina of varying size.

### 3.2. UHF US

One milliliter of NaCl is locally applied to the CAM to enhance acoustic coupling and the transducer is carefully placed onto the CAM. The transducer is gently moved over the engrafted PDX and pressure adjusted until the tissue is focused in the center of the scan (Figure 1b,c). 3D scans are acquired by moving the transducer in a continuous steady sweep over the tissue while always maintaining a consistent parallel hand position to avoid video distortions.

By following the steps described above, videos in high quality were recorded with continuous motion showing the PDX clearly in each frame. Successful scans can be converted into 3D scans as they yield a clear rectangular cross‐section of the scanned area on the CAM (Figure 2a). In contrast, embryonic movements or unsteady handling can lead to distorted edges and reduced scan quality (Figure 2b). Multiple video recordings are therefore practical and improve the quality of the results.

**Figure 2:**
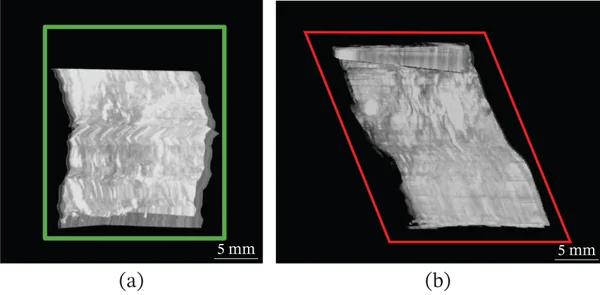
3D scan reconstructions of a PDAC on the CAM from a top‐down perspective. (a) Top view of a high‐quality scan with steady and controlled movement providing an accurate 3D representation. (b) Top view of a distorted scan resulting from unsteady movements during video acquisition.

### 3.3. Software 3D Reconstruction

The 3D sonography system is equipped with two software programs. The first one is responsible for recording videos (Version v4.1#195, software building date September 9, 2024). This software converts the recorded videos into frames. The second component is an analysis software (Version v3.2.1 #182, software building date May 2, 2023) (Figure 3). For optimal reconstruction quality, high‐resolution scans with minimal motion artifacts are recommended (Figure 2a). Representative ultrasonic frames obtained from a high‐quality acquisition are shown in Figure 3c. During image acquisition, the transducer is moved linearly in a single direction across the region of interest. This continuous horizontal movement generates a dense sequence of 2D images, which are automatically reconstructed into a volumetric 3D dataset by the software. The system records spatial *x*, *y*, and *z* coordinates as well as the total distance traveled by the probe, thereby eliminating the need for manual angle adjustment during or after acquisition.

**Figure 3:**
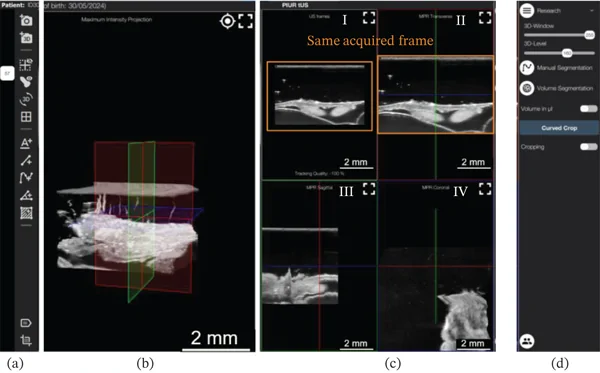
3D imaging Infinity software interface. 3D UHF ultrasonic measurement of PDAC tumors engrafted on the CAM. (a) Options for taking 3D snapshots, viewing the *x*, *y*, and *z* axes. Visualizing the ultrasonic transducer performing 3D rotations and showing or hiding 3D perspectives. (b) 3D reconstruction of a PDAC tumor on the CAM with transverse, coronal, and sagittal planes along the *x*, *y*, and *z* axes that measure the tumor volume. (c) US system in the transverse, sagittal, and coronal planes. The multiplanar reconstruction (MPR) of the acquired 3D US volume is depicted, which demonstrates the coregistration of the current acquisition frame with the three orthogonal reconstruction planes. (i) US frames: the current, native B‐mode frame as acquired live by the transducer during the freehand 3D sweep. (ii) MPR transverse: the transverse multiplanar; in the acquisition and reconstruction configuration used in this study, the transducer′s native scanning plane corresponds to the transverse MPR plane, (i) and (ii) display the same acquired content, shown here at higher on‐screen magnification. (iii) MPR sagittal and (iv) MPR Coronal. (d) Settings.

The reconstructed volume allows multiplanar visualization of the CAM tissue architecture in transverse, sagittal, and coronal planes (Figure 3b,c). These planes can be interactively adjusted by translating or rotating the dataset to refine anatomical orientation. The analysis interface (Figure 3d, right panel) provides multiple quantitative tools for volumetric assessment and structural evaluation. In the present study, the dedicated research analysis module was applied for standardized evaluation of the reconstructed tumor volumes (Figure 3d).

After completing the steps outlined above, the frames are subsequently annotated (Figure 4). When annotating it is essential to review and consider all three dimensions (Figure 4b–e). To achieve high‐quality videos, annotation of the PDAC tumors should be used and the entire annotation process is illustrated in Figure 4. The segmentation process was conducted according to the manufacturer′s instructions ensuring accurate identification of all relevant tissue parts (Figure 4e). After confirming the accuracy of the segmentation of the 3D tumor volume (Figure 4f,g), the measurement can be exported and further processed via software such as Blender. Although the native analysis software provides volumetric measurements, its precision may be limited when assessing very small tumor volumes. To overcome this limitation, the segmented 3D tumor model can be exported (e.g., as STL or OBJ format) into Blender, an open‐source 3D modeling and computational geometry platform. Blender offers advanced mesh analysis tools, precise scaling functions, Boolean operations, and exact volumetric calculations based on closed surface meshes.

**Figure 4:**
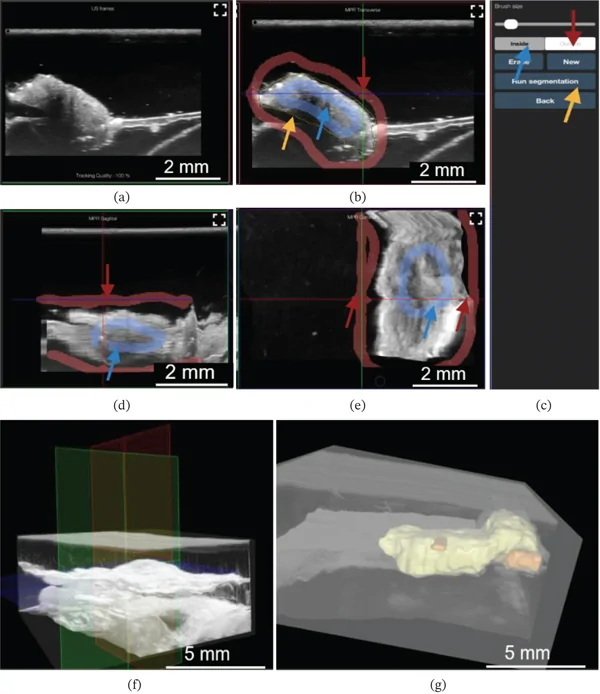
3D US imaging Infinity software research outlay. 3D US measurement of a PDAC tumor engrafted on the CAM. The red arrows represent the user‐defined outer contour, and the blue arrows represent the user‐defined inner contour, whereas the orange arrows represent the software‐generated 3D contour used for volumetric quantification in all panels. (a) US frame overview as indicated while performing the ultrasonic measurement. (b) Transverse plane. (c) Settings. (d) Reconstructed sagittal plane. (e) Reconstructed in the coronal plane. (f,g) Different views of 3D reconstructions of PDAC tumor tissue on the CAM.

For standardized volume measurement, the tumor mesh can be directly compared to a reference cube of known dimensions (1 × 1 × 1 cm; volume = 1  cm^3^) created within Blender. By calibrating the spatial scale and applying Blender′s mesh volume calculation algorithms, an exact tumor volume can be computed independent of the original US software′s internal estimation. This approach enables highly effective volumetric quantification, particularly for small lesions, and enhances reproducibility and cross‐platform validation of 3D US‐derived tumor measurements.

Although the PIUR IMAGING 3D sonography imaging software was originally developed for clinical and diagnostic use and is therefore designed to visualize larger volumes, it can be adapted for visualization of smaller structures in the CAM model. Modifications to the default settings were required to adapt the system to the smaller scale and intricate details of the irregularly shaped engrafted PDAC tumor tissue (Figure 5a,b). These adjustments can be made in the “Personal Settings” file where the interactive segmentation parameters can be tailored to match the ultrasonic transducer resolution and object size.

**Figure 5:**
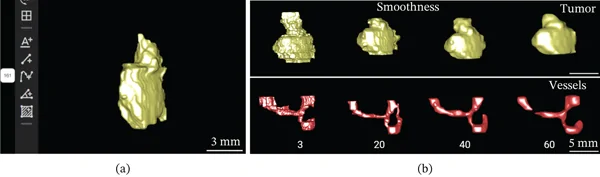
3D US imaging Infinity software reconstructions. 3D US measurement of PDAC tumors engrafted on the CAM. (a) 3D reconstruction without a surrounding US signal. (b) Different options for smoothness fitting of the tumor and blood vessels.

The reliability of ex vivo 3D US for soft‐tissue volumes has previously been demonstrated [13]. To assess its feasibility regarding the in vivo CAM model, phantoms of defined volume (50, 100, and 200 mm^3^) were measured by both 3D UHF‐US and a Keyence VHX‐7000 3D digital microscope (Figure 6 and Videos S1, S2, and S3). Across all volume categories, descriptive Wilcoxon signed‐rank tests did not reveal apparent differences between 3D UHF‐US and digital microscopy measurements. The largest discrepancy was observed in the 50‐mm^3^ category, where digital microscopy tended to underestimate the predefined volume (median ≈37 mm^3^), whereas 3D UHF‐US measurements were closer to the reference value (median ≈50 mm^3^). As an exploratory assessment of agreement with the predefined volumes, separate Bland–Altman analyses were performed for each modality (Figure S4). Both approaches showed overall comparable agreement with the reference values (bias of 3D digital microscopy = −0.56; bias of 3D UHF − US = 2.16). However, digital microscopy demonstrated greater variability, reflected by wider LoA compared with 3D UHF‐US (LoA of 3D digital microscopy = −32.20 to 31.08; LoA of 3D UHF − US = −9.94 to 14.27). These results indicate that 3D US provides volumetric accuracy at least equivalent to established 3D digital microscopy, with a particular advantage at small volumes that are characteristic of CAM‐engrafted tissue. Overall, both methods provide reliable and consistent results across all volume categories (Figures 6 and 7). These findings align with previously published studies that have implemented the routinely used microscopic measurement technique [6]. Additionally, we compared the VHX 7000 series microscope with CT measurements (Figure S3) of human autosomal dominant polycystic kidney tissue. However, smaller tissue samples present a challenge for precise delineation of the target structures, which in turn makes accurate volume determination more challenging.

**Figure 6:**
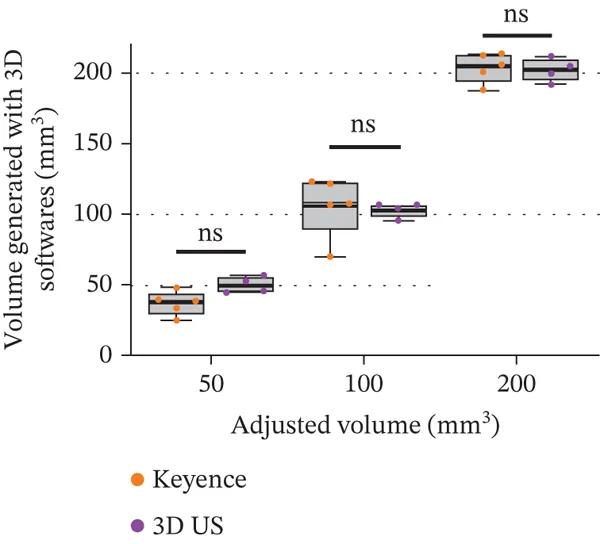
Comparative analysis of 3D UHF ultrasound and Keyence VHX‐7000 3D digital microscopy using phantoms with predefined volume (50, 100, and 200 mm^3^; *n* = 4–5 spheres per category, each measured in duplicates). Box plots show median, interquartile range and individual measurements for each modality. Exploratory Wilcoxon signed‐rank test showed nonsignificant (ns) differences between modalities at every volume. Agreement analysis with reference values was performed by two separate Bland–Altman analyses (Figure S4).

**Figure 7:**
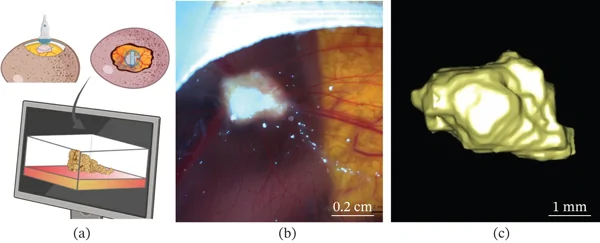
Comparison of a digital photograph and a 3D model after annotation via 3D US software. (a) Schematic representation of the ultrasound scanning setup for the visualization of engrafted tissue in the CAM model (created with http://BioRender.com). (b) Digital photograph taken on the corresponding day. (c) 3D reconstruction of the PDAC tissue generated by the software.

Repeated UHF‐US measurements were performed over several days to test whether a longitudinal measurement approach appeared as feasible. By capturing a series of US videos of the same tumor across multiple days we were able to monitor and evaluate changes in the tumor morphology and structure over time (Figure 8).

**Figure 8:**
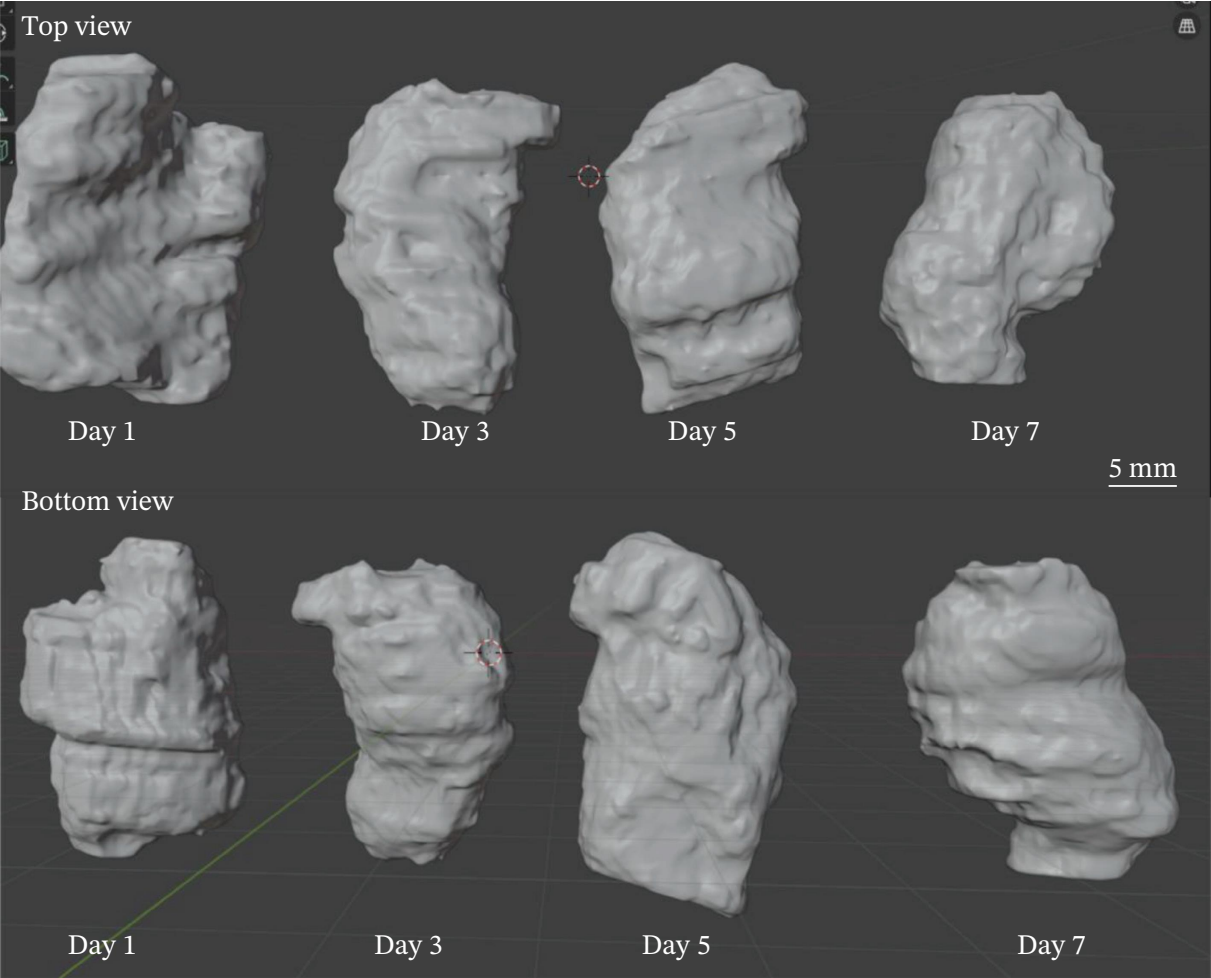
3D reconstructions after annotation via 3D sonography imaging software. The tumors were measured on each other day with the same rotation and top and bottom views.

### 3.4. UHF‐US Scanning Setup and Technique

A significant challenge in 3D US imaging on the CAM is the constrained space that is accessible through the created eggshell window. The mobility of the CAM can occasionally cause the tumor to shift under the eggshell. To mitigate a possible impact in long‐term studies maximizing the window size for optimal transducer movement without damaging the CAM itself is crucial. Maintaining a constant distance between the transducer and the CAM surface with the tumor combined with steady motion during UHF‐US 3D acquisition eliminates possible variations or inaccuracies in 3D reconstruction. By implementing a standardized methodology and set of scanning angles at 90° to the tumor surface area during image acquisition, the transducer is moved linearly in a single direction across the region of interest. The system records spatial *x*, *y*, and *z* coordinates as well as the total distance traveled by the probe, thereby eliminating the need for manual angle adjustment during or after acquisition.

### 3.5. Considerations for 3D Annotations

Accurate annotation is another critical step often requiring the user′s support to assist the software in accurately differentiating between the tumor and the surrounding CAM tissue. Manual intervention is frequently required to refine the automatic detection of tumor boundaries. Each frame can be meticulously reviewed in the sagittal, coronal and transverse planes allowing for a correction of misinterpretations by the software. Errors in annotation such as the inclusion of areas outside the tumor (Figure 9) can be corrected by adding additional markers across all three perspectives (transverse, sagittal and coronal views). Comprehensive annotation in all planes further increases both the accuracy and reproducibility of 3D reconstruction.

**Figure 9:**
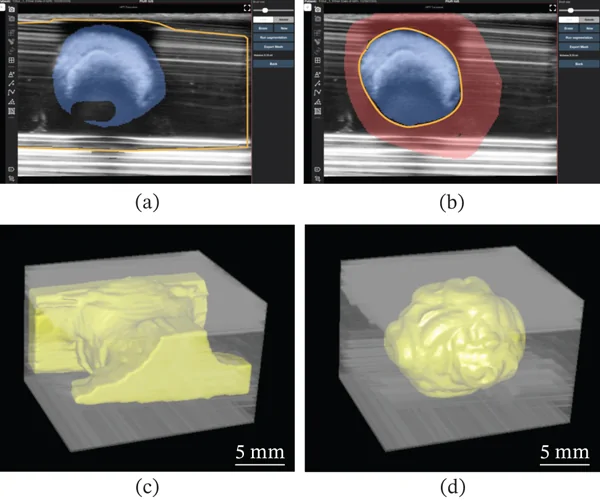
Annotation process. (a) Misidentification of the tumor boundaries by the software (yellow line). The original annotations are shown in blue (representing the interior of the tumor) and red (outlining the external boundary). (b) The software can accurately identify the tumor tissue when further annotation markers are added. (c) The 3D reconstruction of a tumor shows the extent of the software error in boundary detection. (d) The 3D model of (b) reflects the precise shape and boundaries of the tumor.

### 3.6. Comparison of 3D Reconstruction via CT and Digital Microscopy

To further examine volumetric measurements obtained in the CAM model, renal cyst volumes were assessed using both CT imaging and 3D digital microscopy and directly compared. Across all samples (*n* = 10), exploratory statistical analysis did not indicate apparent differences between the two methods (*p* > 0.05, Figure 10a). Paired data points representing identical tissues showed only minor variation between modalities, suggesting overall comparable volumetric results for CT and 3D digital microscopy.

**Figure 10:**
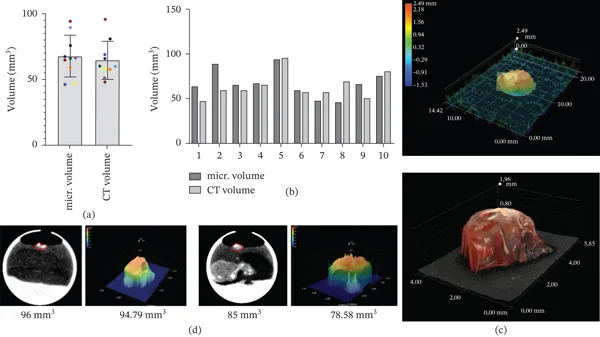
Comparison of the volume of renal cysts measured with 3D digital microscopy and clinical CT scans in the CAM model. CT‐based cyst volumes were determined in the Monaco treatment‐planning software (Table 1, Table 2). After applying a fixed CT DICOM window (width 100, level 40), the cyst was manually delineated slice‐by‐slice across the CT dataset, and the volume of the segmented structure was computed by the software. (a) Volumes of renal cysts were measured in vivo using clinical CT (CT volume) and compared with volumes obtained by 3D digital microscopy (micr. volume) before implantation and 8 days later after excision. Exploratory two‐tailed Wilcoxon matched‐pairs signed‐rank test showed no significant difference (*p* value > 0.05; *n* = 10). Each data point of the same tissue is highlighted with the same color. b) Comparison of individual, 10 corresponding measurements of renal cysts obtained ex vivo via 3D digital microscopy (micr. volume) and in vivo based on clinical CT scans at the beginning of the examination period (*n* = 10 in total). (c) 3D ex vivo volume measurement of a PDAC sample with a Keyence 3D digital microscope. The tumor tissue was excised after engraftment on the CAM for 7 days. (d) Matching volume measurements from a CT scan (left) and a 3D digital microscopy measurement (right) for two different CAM samples.

A detailed comparison of individual cyst volumes revealed a strong concordance between ex vivo measurements obtained via digital microscopy and in vivo CT‐based assessments at the beginning of the observation period (Figure 10b). Although slight deviations were observed in individual samples, no systematic bias toward either method was detected, supporting the interchangeability of both techniques for volumetric analysis in this model.

Representative 3D reconstructions further illustrate the capability of digital microscopy to effectively capture tumor morphology and volume. High‐resolution surface profiling of excised tissue enabled volumetric quantification, as demonstrated for a PDAC sample after 7 days of cultivation on the CAM (Figure 10c). The reconstructed 3D topography allowed for detailed visualization of tumor architecture, including surface irregularities and height distribution. Direct visual and quantitative comparisons between CT‐derived volumes and corresponding 3D microscopy measurements confirmed the close agreement between both modalities (Figure 10d). For matched samples, volumetric differences remained minimal (e.g., 96 vs. 94.79 mm^3^ and 85 vs. 78.58 mm^3^), further supporting the accuracy of optical 3D reconstruction techniques.

## 4. Discussion

Many preclinical cancer studies rely on rodent models [14, 15]. However, the use of relevant orthotopic or genetically engineered animal models is time‐consuming, costly, and often raises ethical concerns. The CAM model resembles a suitable alternative in vivo model that has already gained popularity as a versatile tumor model [5, 13, 16]. As the use of patient‐derived tissues and longitudinal therapeutic studies in the CAM model continues to expand, robust and accurate methods for quantification of tumor growth, morphology and therapy response are of utmost importance to advance this preclinical cancer model. Several imaging techniques, including MRI optical coherence tomography (OCT), micro‐CT, and digital microscopy, have been used to quantify tumor growth and therapeutic responses [17–19]. The reflected‐light digital microscopy approach, which also served as a reference in the present study, has become an established tool for 3D visualizations [6]. Unlike optical‐sectioning techniques such as confocal laser scanning microscopy or light‐sheet fluorescence microscopy, which reconstruct internal 3D structures from fluorescently labeled, optically accessible specimens, reflected‐light digital microscopy is a label‐free, surface‐based technique. It reconstructs the exposed tissue topography using a depth‐from‐defocus (shape‐from‐focus/focus‐variation) approach and derives tumor volume from the reconstructed external surface relative to a reference plane instead of imaging the tissue interior. Consequently, it is well suited for ex vivo surface‐based volumetric analysis but cannot visualize subsurface features such as cystic or necrotic regions, involves terminal tissue excision, and is therefore not applicable to longitudinal in vivo monitoring in the CAM model.

Beyond this, MRI also stands out for its excellent soft tissue contrast and 3D imaging capabilities, making it a valuable tool for embryological studies. However, MRI systems are costly and often require the use of contrast agents or anesthesia to limit movement of the chick embryo, which can introduce undesirable side effects and limit their applicability for longitudinal studies [20], making them impractical for routine experimental setups. Similarly, optical methods such as OCT provide high‐resolution images but lack scanning depths, which makes them unsuitable for the monitoring of larger or more deeply embedded tissue types [21, 22]. Furthermore, these methods typically require the sacrifice of embryos to capture high‐resolution 3D images, which excludes their use in longitudinal studies [23]. Additionally, clinically approved CT scanner was applied in vivo in this study at two timepoints for cross modal comparison rather than as a routine monitoring tool. Likewise, micro‐CT that can generate detailed 3D reconstructions necessitates the fixation of embryos and is thus incompatible with longitudinal in vivo monitoring (Figures S2 and S3).

Alternatively, US represents an attractive imaging approach because it is noninvasive, readily accessible, and well suited for longitudinal studies. However, its use has largely been confined to 2D B‐mode acquisition. Importantly, our own previous research reported UHF‐US as a 2D modality for monitoring renal cysts engrafted on the CAM [7], and comparable approaches have relied on 2D measurements that approximate lesions as regular geometric bodies (e.g., spheres or ellipsoids) and derive volume from one or two orthogonal diameters [24]. Although useful for regularly shaped lesions, such approximations are prone to systematic error when measuring patient derived PDAC tissue, which often exhibit irregular or lobular morphologies and strong internal heterogeneity. To overcome this, the present work integrates a handheld electromagnetic motion sensor with a 71‐MHz UHF transducer, which converts a freehand 2D sweep into a spatially registered volumetric dataset, thereby enabling geometry‐free volume quantification in vivo. To our knowledge, this is the first description of freehand 3D UHF‐US reconstruction applied to solid, patient‐derived tissue on the CAM, extending earlier 2D CAM US approaches from diameter‐based estimation to surface‐mesh‐based volumetry that additionally resolves internal morphological features such as cystic and necrotic regions. Importantly, 3D UHF‐US enables reconstruction of tumor structures and provides volume measurements as demonstrated in our comparative analysis with 3D digital microscopy and CT (Figures 6, 10, and S4). A key advantage of 3D UHF‐US is the ability to visualize the tumor morphology in detail including tumor spheroids, cysts, and necrotic areas thereby providing insights into the structural organization of tumors. Furthermore, by eliminating the need for contrast agents and enabling continuous in vivo monitoring, 3D UHF‐US also enables long‐term visualization of tumor development and provides a framework for potential assessment of therapeutic response in future studies. Notably, several clinical studies have documented the use of 3D US for quantitative assessment involving patients with conditions such as abdominal aortic aneurysms, cerebrovascular disorders, and Parkinson′s disease [25–27]. Lastly, the rapid acquisition time (typically 5–10 min per CAM model), together with its noninvasive and longitudinal imaging capabilities, makes 3D UHF‐US a practical tool for routine volumetric and morphological assessments in the CAM model. Nonetheless, no vascular or angiogenic quantification was performed in the present work as dedicated vascular read‐outs would require complementary modalities such as color/power Doppler or contrast‐enhanced and super‐resolution US, which were beyond the scope of the current study. Should such modalities be integrated, the spatial resolution and repeatability of UHF‐US could in principle allow longitudinal monitoring of micro vessel formation without sacrificing the embryo or administering contrast agents, in contrast to MRI or micro‐CT.

Although this study showcases the feasibility of freehand 3D UHF‐US reconstruction in the CAM model, certain methodological limitations should be considered. First, image acquisition remains operator‐dependent, requiring a steady freehand sweep and consistent transducer positioning. In addition, spontaneous movement by the embryo may introduce motion artifacts and necessitate repeated acquisitions. Second, although fully automated segmentation is under development, this study had to rely on manual review and correction of the annotations, limiting throughput capacity and requiring a trained operator. Third, the maximum penetration depth of approximately 10 mm. Finally, comparative analyses were limited to small sample sizes of geometric phantoms and renal cyst tissue, using 3D digital microscopy and clinical CT as references, which restricted statistical testing to descriptive purposes only. Therefore, future studies should extend to larger biological cohorts, additional tumor entities, and therapy‐response settings. Together with the incorporation of additional reference approaches, this will further develop the methodology.

Beyond static volumetric measurement of PDAC, several extensions of this approach are possible. The noninvasiveness of 3D UHF‐US makes it well suited for longitudinal imaging of tissues engrafted on the CAM, enabling repeated assessment of the same graft over time. In addition to monitoring growth kinetics, this approach could facilitate longitudinal evaluation of therapy response by tracking treatment‐induced changes such as tumor regression, growth delay, and the development of cystic or necrotic regions following topical or intravascular drug administration. Building on this capability, integration with Doppler US or, where feasible, contrast‐enhanced and super‐resolution US could provide complementary information on tumor angiogenesis and vascular remodeling, alongside established readouts such as LASCA [8]. Beyond PDAC, the workflow could also be applied to other PDX, cCSC‐derived tumor spheroids, and nononcological engrafted tissues, e.g. renal cystic tissue. More broadly, this approach may support personalized therapy assessment in alternative PDX models, while reducing the need for rodent‐based experiments, aligning with the 3R‐principles. Finally, automated learning‐based segmentation and integration with complementary modalities, including digital microscopy, could streamline image analysis, reduce operator dependence, and facilitate standardized, higher‐throughput therapy screening in the CAM model.

## 5. Conclusions

The 3D UHF‐US is a versatile 3D imaging method for the measurement of PDAC tumor samples engrafted on the CAM, supported by comparative evaluation against 3D digital microscopy measurements. It resembles a noninvasive, rapid measurement method for the assessment of tumor morphology and volumetric quantification that overcomes many limitations such as a complicated setup, high costs, and potential harm by injected agents or ionizing radiation. Further studies are necessary to determine the applicability of this approach for other human‐derived tissue types or tumor entities and potential therapy testing with the CAM model.

## Author Contributions

Andreas Ettner‐Sitter: conceptualization, methodology, investigation, formal analysis, data curation, writing‐original draft, and writing‐review and editing; Jonas Kuenzel: methodology, investigation, formal analysis, data curation, and writing‐original draft; Mia Heidenreich: methodology, investigation, formal analysis, data curation, and writing‐original draft; Lina Mahl: formal analysis, data curation, writing‐original draft, and writing‐review and editing; Luise Pohnert: methodology, formal analysis, validation, writing‐original draft, and writing‐review and editing; Dharini Srinivasan: validation, formal analysis, supervision, and writing‐review and editing; Eric Pion: methodology, formal analysis, writing‐original draft, and writing‐review and editing; Robert Bauer: methodology, resources, and writing‐review and editing; Dieter Fuchs: methodology, resources, and writing‐review and editing; Benedikt J. Wagner: methodology, formal analysis, funding, writing‐original draft, and writing‐review and editing; Christian Gröger: methodology, formal analysis, and writing‐review and editing; Felix Steger: methodology, formal analysis, and writing‐review and editing; Oliver Kölbl: methodology, formal analysis, and writing‐review and editing; Thiha Aung: methodology, resources, project administration, and writing‐review and editing; Christina Hackl: methodology, resources, project administration, and writing‐review and editing; Silke Haerteis: conceptualization, investigation, supervision, project administration, funding, writing‐original draft, and writing‐review and editing.

## Funding

No funding was received for this manuscript. Open Access funding enabled and organized by Projekt DEAL.

## Disclosure

All the authors agree to take accountability for the accuracy and integrity of all aspects of the work. All authors have read and approved the final manuscript. All authors take full responsibility for all content presented in this manuscript.

## Ethics Statement

All procedures performed in the study were in accordance with the ethical guidelines of the “World Medical Association Declaration of Helsinki ‐ Ethical Principles for Medical Research Involving Human Subjects.” Approval from the ethics committee of the University Hospital Regensburg was obtained (Ethics Approval Number 20‐1989‐101).

## Consent

Written informed consent was obtained from the patients for publication. A copy of the written consent is available for review by the editor in chief of this journal upon request.

## Conflicts of Interest

The authors declare no conflicts of interest.

Robert Bauer is cofounder and managing director of PIUR IMAGING GmbH, which developed the 3D ultrasound reconstruction software used in this study. PIUR IMAGING GmbH provided technical support and software adaptations for the high‐frequency transducers used. No financial sponsorship, free equipment, or other material support was provided. The imaging system was purchased by the University of Regensburg under standard commercial terms. Dieter Fuchs is employed by FUJIFILM VisualSonics. He was not involved in any form of data analysis, writing of the manuscript that exceeded the methodology itself, and any conclusions drawn from the experiments.

## Supporting information


**Supporting Information** Additional supporting information can be found online in the Supporting Information section. Figure S1: Stationary mount for the UHF ultrasound transducer. Figure S2: Representative 3D ultrasound measurements of three tumor samples obtained with the 3D sonography tracking system. Figure S3: CT imaging planes during examination of a CAM model. Figure S4: Bland–Altman analyses of 3D UHF ultrasound and Keyence VHX‐7000 3D digital microscopy using volume phantoms. Video S1: 3D UHF ultrasound scan of a 50‐mm^3^ phantom. Video S2: 3D UHF ultrasound scan of a 100‐mm^3^ phantom. Video S3: 3D UHF ultrasound scan of a 200 mm^3^ phantom.

## Data Availability

The data that support the findings of this study are available from the corresponding author upon reasonable request.
