# Supplementary material for: High‐Resolution Imaging via UHF‐US–Based 3D Reconstruction in a 3D In Vivo Tumor Model
Source: Int J Biomed Imaging. 2026 Oct 26;2026:8763250. doi: 10.1155/ijbi/8763250 (PMC13710809; doi:10.1155/ijbi/8763250)
Supplement: Supplementary file 1 [file IJBI-2026-8763250-s001.zip › 5_Ettner_et al Revision_Supplements.docx]

Supplementary Figures

High-resolution imaging via UHF-US based 3D reconstruction in a 3D *in vivo* tumor model

Andreas Ettner-Sitter^1,2^, Jonas Kuenzel^1^, Mia Heidenreich^3^, Lina Mahl^1^, Luise Pohnert^1^, Dharini Srinivasan^1^, Eric Pion^1,7^, Robert Bauer^4^, Dieter Fuchs^5^, Benedikt J. Wagner^2^, Christian Gröger^6^, Felix Steger^6^, Oliver Kölbl^6^, Thiha Aung^7^, Christina Hackl^2^ and Silke Haerteis^1^

1 Andreas Ettner-Sitter: andreas.ettner-sitter@ur.de orchid: 0009-0008-6443-2597
2 Jonas Kuenzel: Jonas.Kuenzel@stud.uni-regensburg.de
3 Mia Heidenreich: mia.heidenreich@tum.de
4 Lina Mahl: lina.mahl@me.com
5 Luise Pohnert: Luise.Pohnert@stud.uni-regensburg.de
6 Dharini Srinivasan: dharini.srinivasan@vkl.uni-regensburg.de
7 Eric Pion: Eric.Pion@vkl.uni-regensburg.de
8 Robert Bauer: Bauer@piurimaging.com
9 Dieter Fuchs: dieter.fuchs@fujifilm.com
10 Benedikt J. Wagner: Benedikt.wagner@ukr.de
11 Christian Gröger: c.groeger@ukr.de
12 Felix Steger: f.steger@ukr.de
13 Oliver Kölbl: o.koelbl@ukr.de
14 Thiha Aung: Thiha.aung@th-deg.de
15 Christina Hackl: Christina.hackl@ukr.de
16 Silke Haerteis: Silke.haerteis@ur.de *

**Corresponding author:**

**Prof. Dr. Silke Härteis**

Institute for Molecular and Cellular Anatomy,

Fakultät für Biologie und Vorklinische Medizin

Universität Regensburg

Universitätsstraße 31

D-93053 Regensburg

**Email: Silke.haerteis@ur.de**

1. Institute for Molecular and Cellular Anatomy, University of Regensburg, Regensburg, Germany
2. Department of Surgery, University Hospital Regensburg, Regensburg, Germany
3. TUM School of Life Sciences, Technical University of Munich, Freising, Germany
4. PIUR IMAGING GmbH, Vienna, Austria
5. FUJIFILM VisualSonics Inc., Amsterdam, The Netherlands
6. Department of Radiotherapy, University Hospital Regensburg, Regensburg, Germany
7. Faculty of Applied Healthcare Science, Deggendorf Institute of Technology, Deggendorf, Germany

Supplementary files

The following supporting information can be downloaded at: Figure S1: Stationary mount for the UHF ultrasound transducer; Figure S2: Representative 3D ultrasound measurements of three tumor samples obtained with the 3D sonography tracking system; Figure S3: CT imaging planes during examination of a CAM model; Figure S4: Bland-Altman analyses of 3D UHF ultrasound and Keyence VHX-7000 3D digital microscopy using volume phantoms; Video S1: 3D UHF ultrasound scan of a 50 mm³ phantom; Video S2: 3D UHF ultrasound scan of a 100 mm³ phantom; Video S3: 3D UHF ultrasound scan of a 200 mm³ phantom.

Supplemental Figure 1: Stationary mount a) Picture of the entire setup with the stationary mount and the UHF transducer. b) Mount arm with crank adjustable Z axis. c) Mount table with the crank adjustable X- and Y-axes.

Supplemental Figure 2: 3D ultrasonic measurements with the 3D sonography imaging tracking system. Representative 3D tumor measurements of three different tumor samples. (created with BioRender.com).

Supplemental Figure 3: Imaging planes of the CT device during the examination of a CAM model (insets: CAM model with engrafted tissue). The small images show a kidney cyst engrafted onto the CAM on day 10 after incubation.

Supplemental Figure 4: Separate Bland-Altman analyses of 3D UHF ultrasound and Keyence VHX-7000 3D digital microscopy using phantoms of predefined volume (50, 100, and 200 mm³; n = 4 to 5 spheres per category, each measured in duplicates). Each point represents the difference between independent measurements and expected value plotted against their mean. Mean bias (solid line) and 95% limits of agreement (LoA; dashed lines) are indicated for each modality.

**Supplementary Video**

Videos submitted as part of the supplementary files.
